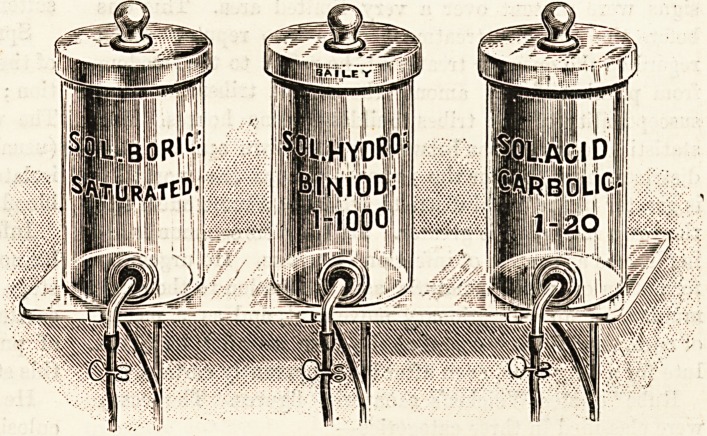# Some Hospital Appliances and Ward Furniture at Messrs. Bailey's New Premises

**Published:** 1901-03-16

**Authors:** 


### THE INSTITUTIONAL WORKSHOP.

### SOME HOSPITAL APPLIANCES AND WARD FURNITURE AT MESSRS. BAILEY'S NEW PREMISES.

MESSRS. BAILEY AND SON, of 38 Oxford Street, have recently opened a showroom for hospital furniture and appliances at 2 Rathbone Place, where examples of their well-known manufactures may be seen to great advantage, as well as some new designs. Some of these articles have been waiting for some time at 38 Oxford Street, but owing to lack of space they have only been shown when inquired for. The showroom in Rathbone Place is roomy and not over-crowded; and intending purchasers for hospitals or private patients should make a visit of inspection to it before deciding on what to buy. There is a great choice of white-enamelled iron and plate - glass furniture, including several varieties of aseptic ward tables of different shapes and sizes. The best of these is made with rounded iron legs, polished plate - glass shelves, and brass castors with indiarubber tyres on which the table runs readily and silently. The glass shelves are made to fit loosely into the rims, so as to be easily lifted out for cleaning. A special bed-foot table is made for typhoid cases: by means of rubber castors it moves easily along the floor of the ward, it has round iron legs, three plate-glass shelves, and a towel-rail at each end. The size is 30 inches long by 12 inches wide, and the height is 35 inches. A number of these tables has recently been

The jug should be placed on the floor, not as shown in woodcut.

supplied to St. George's Hospital. Dressing waggons, bandage cabinets, instrument cases, and ward lockers may be had in the same material, *i.e.* white-enamelled iron and plate glass. Great care has been taken to make the cabinets for instruments as impervious to dust and germs as possible; by means of a rim which runs along the door-opening, particles of dust which settle on the edge of the door are prevented from getting inside. One of these cases is of glass on each side, top, door, and back the fittings are nickel-plated. Another has glass door, sides, and top, with iron back. In size, they are from 18 inches high, by 13½ inches wide and 8 inches deep, to 5 feet 8 inches high by 3 feet wide and 1 foot 4 inches deep, but any size can be had to order.

The ward locker is a recent development in this style of furniture; it has already been supplied to several hospitals. There is a drawer above and cupboard below, each fitted with a ground-glass shelf; the locker runs smoothly and quietly on rubber tyres, and is therefore easily moved for cleaning purposes. It may be had with three shelves and a drawer, but this has no cupboard, and is on stump legs. A useful solution shelf is also shown; it is of thick glass with polished edges, on brass nickel-plated brackets for fixing to the wall; the jars are engraved in clear red type, which should prevent any possible mistake as to their contents. This has been supplied to the surgeon of the Gloucester Hospital for children.

The accompanying illustration shows a portable irrigator-stand; it is made to carry a 2-gallon glass jar; there is a 6-foot length of indiarubber tubing, and the stand is mounted on indiarubber-tyred castors; it is easy to move it along the floor of the ward with one hand, and thus to save both time and labour.

Among sterilisers may be noticed one for general use in hospitals; it is double copper-jacketed, the outer iron case is packed with asbestos, and the inner vessel varies in size from 12 inches deep by 8 inches in diameter, to 19½ inches deep by 12½ inches in diameter. The box in which the dressings are placed is nickel-plated, a glass tube shows the height of the water, and a thermometer registers the degree of heat. This steriliser is being supplied to several hospitals.

One of the most simple and yet useful objects is that shown in the accompanying illustration—a light and, portable "expanding hoop cradle." It was designed by Mrs. Alfred Paine, of Bedford, and is on the principle of the "cage" in croquet. It folds flat and hangs on a nail when not in use, and may be employed not only for removing the weight of the bedclothes, but, by the addition of a flannel or mackintosh square, for the support of hot-water bottles or ice over the injured limb. By this means the temperature can be raised from 30° to 40°, or lowered from 10° to 15°. On account of its lightness and portability, this cradle would be especially useful to a district nurse in the country.

Perhaps more especially suited to the needs of private patients is the "Gemidea" bed-table. It is of oak, on an iron stand of which the foot goes under the bed. By means of a strong spring the table can be adjusted for reading, &c., in either a horizontal or sloping position.

The above does not pretend to exhaust the list of articles on view at Messrs. Bailey's new premises, where a great variety of glass and rubber goods may be seen, as well as the well-known surgical instruments, &c., detailed in their catalogue, now in its (1900-1) seventh edition.

## Figures and Tables

**Figure f1:**
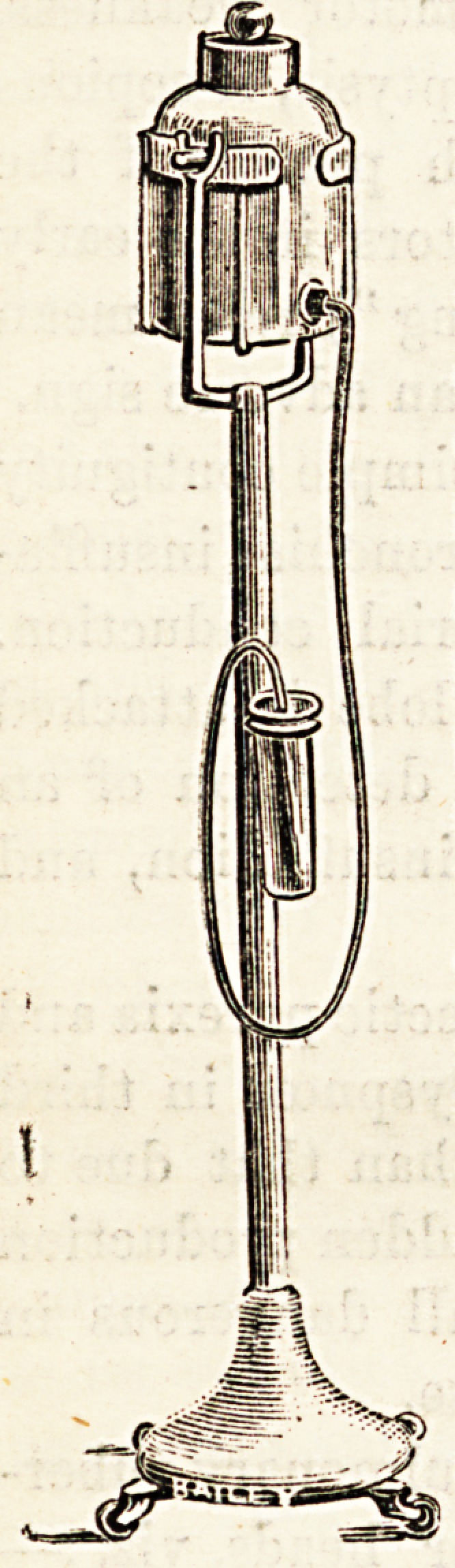


**Figure f2:**
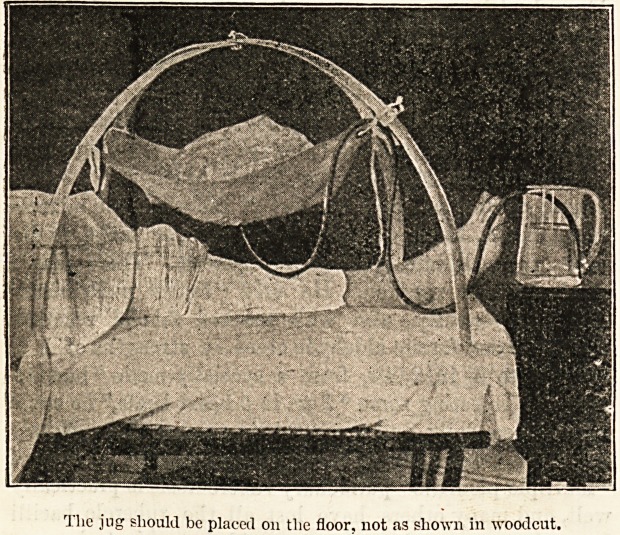


**Figure f3:**